# CircBRIP1: a plasma diagnostic marker for non-small-cell lung cancer

**DOI:** 10.1007/s00432-023-05558-5

**Published:** 2024-02-08

**Authors:** Xinfeng Fan, Qi Zhang, Shiyi Qin, Shaoqing Ju

**Affiliations:** 1 Department of Laboratory Medicine, Affiliated Hospital of Nantong University, Medical School of Nantong University, Nantong, 226001 Jiangsu China; 2 Medical School of Nantong University, Nantong University, Nantong, China; 3 Research Center of Clinical Medicine, Affiliated Hospital of Nantong University, Nantong, 226001 Jiangsu China; 4 Present Address: Department of Laboratory Medicine, Affiliated Hospital of Nantong University, No. 20, Xisi Road, Nantong, 226001 Jiangsu China

**Keywords:** Non-small-cell lung cancer, circBRIP1, Plasma biomarker, Diagnosis, Prognosis

## Abstract

**Background:**

Circular RNA (circRNA), which has been demonstrated in studies to be abundantly prevalent in tumor cells and bodily fluids and to play a significant role in tumors, has the potential for biological markers to be used to assist tumor diagnosis. This study mainly discusses the potential of circBRIP1 as a biomarker for diagnosing non-small-cell lung cancer (NSCLC).

**Methods:**

First, high-throughput sequencing screened the differentially expressed circBRIP1, and real-time fluorescence quantitative PCR (qRT-PCR) verified its expression in NSCLC. Next, sanger sequencing, agarose gel electrophoresis, RNase R assay, and fluorescence in situ hybridization (FISH) were used to verify its molecular characteristics. The diagnostic value was analyzed by the subject operating characteristic curve (ROC), and the cardinality test was analyzed for correlation with clinicopathological parameters. Finally, we tentatively predicted the downstream miRNA- or RNA-binding protein that may bind to circBRIP1.

**Results:**

CircBRIP1 is highly expressed in NSCLC tissues, cells and plasma with good specificity and stability. CircBRIP1 not only can well-distinguish NSCLC patients from benign pulmonary diseases (BPD) patients, healthy individuals and small cell lung cancer (SCLC) patients, but it also has some potential for dynamic monitoring. Combined with the analysis of clinicopathological data, the high level of circRNA expression was related to the degree of tumor differentiation, TNM stage, T stage, lymph node metastasis and distal metastasis in NSCLC patients. In addition, circBRIP1 has a high diagnostic value.

**Conclusions:**

Plasma circBRIP1 is significantly overexpressed in NSCLC patients. It can be used as a sensitive biomarker with unique value for early diagnosis, tumor development and prognosis detection.

**Supplementary Information:**

The online version contains supplementary material available at 10.1007/s00432-023-05558-5.

## Introduction

Lung cancer is the leading cause of cancer-related deaths, with non-small-cell lung cancer (NSCLC), one of the most frequent types of lung cancer, having a poor prognosis (Ruiz-Cordero and Devine 2020). Despite advances in NSCLC treatment, the average 5-year survival rate is only 15% and early metastases are becoming more common (Sung et al. 2021). Clinical studies have found that due to the lack of early diagnosis and clinical features, more than 70% of NSCLC patients are already in mid-to-late-stage lung cancer when the diagnosis was made (Govindan et al. 2008), and have lost the best time for surgical and multidisciplinary radical treatment, which is difficult to treat and has a poor prognosis (Gridelli et al. 2015). Therefore, the key to improving the treatment outcome of NSCLC is improving the early diagnosis. Low-dose computed tomography (LDCT) screening, percutaneous biopsy, positron emission tomography (PET), and liquid biopsy are all used for early NSCLC screening. However, in the clinic, ldct has a significant risk of false positives (Goebel et al. 2019). In addition, invasive examinations or imaging have disadvantages, such as not being able to distinguish well between benign and malignant nodules (McWilliams et al. 2006). Traditional testing of serum tumor biomarkers has become an important diagnostic tool for patients with NSCLC, because liquid biopsies are non-invasive and can be monitored in real time.

Circular RNAs (circRNAs) are a specific type of non-coding RNAs that do not have a 5′ cap and a 3′ poly(A) tail and are covalently bonded to form a closed loop structure (Szabo and Salzman 2016). It has been shown to be abundantly present in the cytoplasm of eukaryotic cells, with species-, tissue-, and time-specific expression levels (Salzman et al. 2013; Maass et al. 2017; Xia et al. 2017). In addition, circRNAs are not affected by RNA exonucleases, more steadily expressed, and less susceptible to degradation than traditional linear RNAs (Jeck and Sharpless 2014). These properties allow circRNA to be stably detected in body fluids (Kristensen et al. 2022), suggesting that circRNA has the potential to become a molecular marker.

CircRNAs have long been considered to be byproducts with no regulatory function arising from abnormal shearing (Huang et al. 2017). However, due to the rapid development of high-throughput sequencing and bioinformatic analysis technologies, circRNAs have been discovered in large numbers and a few of their functions have been investigated (Kristensen et al. 2019). Recently, studies of circRNAs associated with tumors have shown that they are aberrantly expressed in several common human tumors and have potential as a biomarker for tumor diagnosis. For instance, the low expression of the marker hsa _circ _0087776 in the serum of individuals with multiple myelom may make it more effective to diagnose the disease and assess the prognosis (Gong et al. 2021). Yuan et al.’s data demonstrate that circRNA_102231 is significantly up-regulated in gastric cancer tissue and plasma samples and acts as a potential biomarker for gastric cancer patients (Yuan et al. 2021). In addition, it has been discovered to be clearly correlated with tumor cell proliferation, metastasis, and apoptosis, to control the growth of tumors, and to be employed in the prognosis, diagnosis, and therapy of a number of illnesses (Chen and Shan 2021). CircIFI30 high expression was positively linked with clinical TNM stage, pathological grading and poor prognosis in triple-negative breast cancer patients (Xing et al. 2020). Hsa_circ_0001451 was strongly decreased in kidney cancer tissues, which significantly increased the proliferation of kidney cancer cells, and its differential expression was related to the differentiation of renal cell carcinoma (Wang et al. 2018a). Hsa_circ_0001445 induced apoptosis and reduced hepatocellular carcinoma cell growth, migration, and invasion (Yu et al. 2018). These findings demonstrated the potential of circRNA as an emerging biomarker for cancer identification and targeted treatment.

In this study, we first determined significant differences in circBRIP1 expression in NSCLC tissues compared to the surrounding normal tissues by high-throughput sequencing, then confirmed its high expression in cell and plasma samples, evaluated its clinical diagnostic value, examined its correlation with clinicopathological characteristics, and explored its ability to serve as a novel molecular diagnostic marker.

## Materials and methods

### Collection of plasma and tissue samples

The Affiliated Hospital of Nantong University's Ethics Committee gave its approval to this study (Ethics Review Report No.: 2018-L055). The plasma samples collected in this study were from the clinical laboratory of the Affiliated Hospital of Nantong University, including 100 NSCLC patients, 40 benign pulmonary diseases (BPD) patients, 40 small cell lung cancer (SCLC) patients, and 100 healthy subjects. Sixteen pairs of cancerous tissues and surrounding normal tissues were collected. The distance between the paracancerous tissue and the tumor tissue was 3 cm in NSCLC patients, and H&E staining confirmed that there was no tumor infiltration in the paracancerous tissue. All of the patients had clinicopathological diagnoses and received no anticancer therapy. Prior to processing, all samples were kept in a − 80 °C refrigerator. Other clinicopathological information was collected from medical records. All participants obtained informed consent and agreed to publication prior to the clinical trial.

### RNA extraction, cDNA synthesis and quantitative real-time polymerase chain reaction (qRT-PCR)

In accordance with the manufacturer's instructions, TRIzol reagent (Invitrogen, Karlsruhe, Germany) was used to extract RNA from all of the plasma, tissues, and cells used in this experiment, and the extracted total RNA was reverse transcribed into cDNA using a reverse transcription kit (Thermo Fisher Scientific, Waltham, MA, USA). The reverse transcription system include 4 µl 5 × Reaction Buffer, 2 µl 10 mM deoxyribonucleotide triphosphate (dNTPs), 1 µl Oligo (dT) Primer, 1 µl RNase inhibitor (20 U/µl), 1 µl Reverse Transcriptase (200 U/µl), and 11 µl RNA solution. The Roche Light Cycler 480 (Roche, Switzerland) was used to carry out qRT-PCR to identify the expression levels of circBRIP1 in various samples. 10µL the SYBR Green I Mix (Roche), 1 µL primer, 5 µL cDNA, and 4 µL enzyme-free water made up the qRT-PCR reaction system. The PCR cycle consisted of activating enzyme at 95 °C for 10 min, denaturing at 95 °C for 15 s, annealing at 60 °C for 30 s, and then collecting fluorescence information at 80 °C. The 2^−△△CT^ technique was used to examine the PCR data. The forward primer sequence of circBRIP1 is 5 CCAAGAGATGAAGTGGGAGCAC-3, and the reverse primer is 5-AATATCTGAAAAGGCCTTGTAAG-3, synthesized by Suzhou Ribo Biological Co. Prior to Sanger sequencing an agarose gel electrophoresis, PCR products were kept in a refrigerator at − 20 °C.

### Cell culture

Human bronchial-like epithelial cells (HBE) and NSCLC cell lines (NCI-H1299, A549, PC-9, NCI-H226) were purchased from the Cell Bank of the Chinese Academy of Sciences (Shanghai, China). All cells were cultured in RPMI 1640 or DEME media (Gibco, USA) supplemented with 1% penicillin and streptomycin (HyClone, Logan, UT, USA) and 10% fetal bovine serum (Gibco, Waltham, MA, USA). The culturing environment is an incubator set at 37 °C with 5% CO2 humidification.

### Room temperature placement and repeated freezing and thawing

The patient's mixed plasma was kept at room temperature for 0, 6, 12, 18 and 24 h, while the second mixed plasma was repeatedly freeze–thawed for 0, 1, 3, 5 and 10 times to extract plasma RNA and detect the expression of circBRIP1 to verify its stability. Repeat this three times.

### Linearity validation and cell secretion experiments

Patient plasma cDNA was diluted according to gradients 10^1^, 10^2^, 10^3^, 10^4^ and 10^5^, and qRT-PCR was used to identify the linear expression of circBRIP.

The cell supernatants of PC-9, NCI-H1299 and HBE on days 1, 3, 5 and 7 were collected, respectively, and RNA was extracted to detect the expression of circBRIP1.

### Actinomycin D and RNase R experiments

1000 mg/ml of actinomycin D was diluted to 2.5 μg/ml with 1640 complete medium. After NCI-H1299 was incubated in six-well plates for 24–48 h, 2 ml of the above complete medium containing actinomycin D was placed in each well and cultured for 0, 2, 4, 8, 12, and 24 h, respectively. Ribonuclease R (RNase R) was purchased from Geneseed Biotech Co., Ltd. (Guangzhou, China). RNase R was added to the total RNA extracted from NCI-H1299, followed by a digestion reaction system containing 5 μl of 10 × reaction buffer. Immediately afterwards, the reaction mixture was incubated at 37 °C for 30 min and at 70 °C for 10 min. The reverse transcription reaction was then performed.

### Agarose gel electrophoresis

Dilute 50 × TAE to 1 × , configure a 2% agarose gel, and then immediately add EB dye and mix. After cooling, 5 μl of qRT-PCR product and 1 μl of loading buffer were added and mixed, and electrophoresis was performed at 110v–120v for about 40 min. The gel imager was used to view the outcomes.

### Fish (fluorescence in situ hybridization)

According to the manufacturer's instructions, a fluorescent in situ hybridization kit (RiboBio, Suzhou, China) was used to detect the localization of circBRIP1 in cells. After culturing the cells and spreading 24-well plates, 300 µl of pre-hybridization solution was added to each well and closed at 37 °C for 30 min. After discarding the pre-hybridization solution, 300 µl of hybridization solution containing the probe (RiboBio, Suzhou, China) was added under light-proof conditions and incubated at 37 °C overnight. Then each well was rinsed with Wash I, II, III and PBS in turn and photographed by fluorescence microscopy (Olympus, Tokyo, Japan) after adding anti-fluorescence quenching blocking solution (Antifade Mounting Medium with DAPI) (Beyotime, Jiangsu, China).

### Bioinformation analysis

Gene ontology (GO) enrichment analysis of differentially expressed genes was implemented using ClusterProfiler (v3.12.0) to describe the molecular function (MF), cellular components (CC), and biological processes (BP). The KEGG (Kyoto Encyclopedia of Genes and Genomes) pathway for differentially expressed gene enrichment was examined using KOBAS v3.0 software. Plots were made using ggplot2 v3.4.1. *P* values < 0.05 were considered significantly enriched for differentially expressed genes.

### Data analysis

All statistical data in this research were analyzed using SPSS 20.0 (IBM SPSS Statistics, Chicago, USA) software and plotted using GraphPad Prism V.9.00 (GraphPad Software Inc., California, USA). In addition, all data were shown as the mean ± standard deviation (SD) of the outcomes from three separate examinations. As necessary, differences between groups were examined using paired *t* tests and one-way analysis of variance (ANOVA), and the relationship between circBRIP1 and clinicopathological variables was examined using Chi-square tests. Non-parametric analysis was performed to obtain the subject operating characteristic curve (ROC) and area under curve (AUC). Bivariate logistic regression was used to analyze the diagnostic potential of circBRIP1, CEA, SCC, and Cyfra21-1. The critical value was determined using the Uden index (Uden index = sensitivity + specificity—1). P < 0.05 were considered statistically significant differences.

## Results

### CircBRIP1 expression profile in NSCLC tissues, cells and plasma

**Fig. 1:**
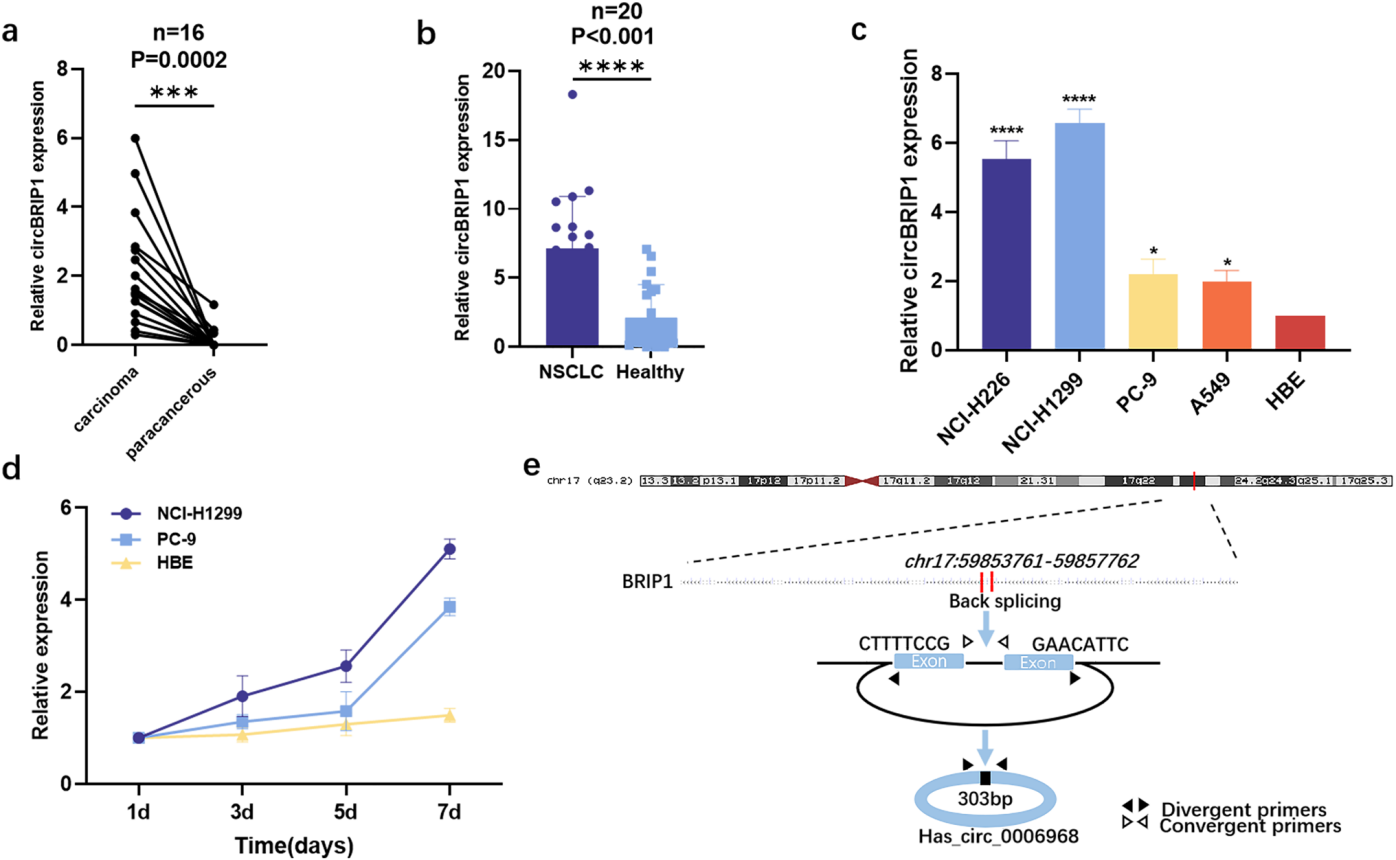
Initial screening and sourcing of circBRIP1. **a** 16 pairs of tissue samples to confirm the upregulation of circBRIP1 (*n* = 16);** b** 20 pairs of plasma samples to confirm the upregulation of circBRIP1 (*n* = 20); **c** 5 cells lines of NSCLC to certify the upregulation of circBRIP1(*n* = 5); **d** CircBRIP1 was secreted into the culture medium by NCI-H1299 and PC-9 cells in a time-dependent manner compared to HBE; **e** location and origin of circBRIP1

High-throughput sequencing of three pairs of cancer tissues and adjacent non-tumor tissues from NSCLC patients was carried out to assess the differential expression of circRNAs, and the sequencing results were visualized by volcano plot and heat map (supplementary images). We selected 6 molecules from the difference multiple log FC > 2.0, *P* < 0.05 for preliminary screening. We found that except circBRIP1, other molecules had problems such as bimodal dissolution curve and unstable expression level, so circBRIP1 was initially selected. First, the expression level of circBRIP1 was initially verified in 16 pairs of NSCLC tissues. In comparison with the equivalent paracancerous tissues, the expression level of circBRIP1 in cancer tissues was considerably increased (Fig. 1a). Second, 20 NSCLC patients had plasma circBRIP1 expression levels that were considerably greater than in 20 healthy individuals (Fig. 1b). Finally, the results of qRT-PCR demonstrated that circBRIP1 expression levels were elevated in NSCLC cell lines (NCI-H226, NCI-H1299, PC-9, A549) compared to healthy bronchial-like epithelial cell lines (HBE) and were most significantly elevated in NCI-H1299 (Fig. 1c). In addition, the expression level of circBRIP1 in the supernatant of NCI-H1299 and PC-9 cells was elevated with increasing culture time compared to HBE, suggesting that circBRIP1 may be secreted by NSCLC cells and is associated with the presence of NSCLC (Fig. 1d). The Circbank database (http://www.circbank.cn/index.html) showed that circBRIP1 was located on chromosome 17 with a mature transcript size of 303 bp (Fig. 1e). In summary, circBRIP1 was greatly expressed in tissues, cells and plasma, which is consistent with the sequencing results and allows for follow-up studies.

### Molecular characterization of circBRIP1 and methodological evaluation of its detection level

**Fig. 2:**
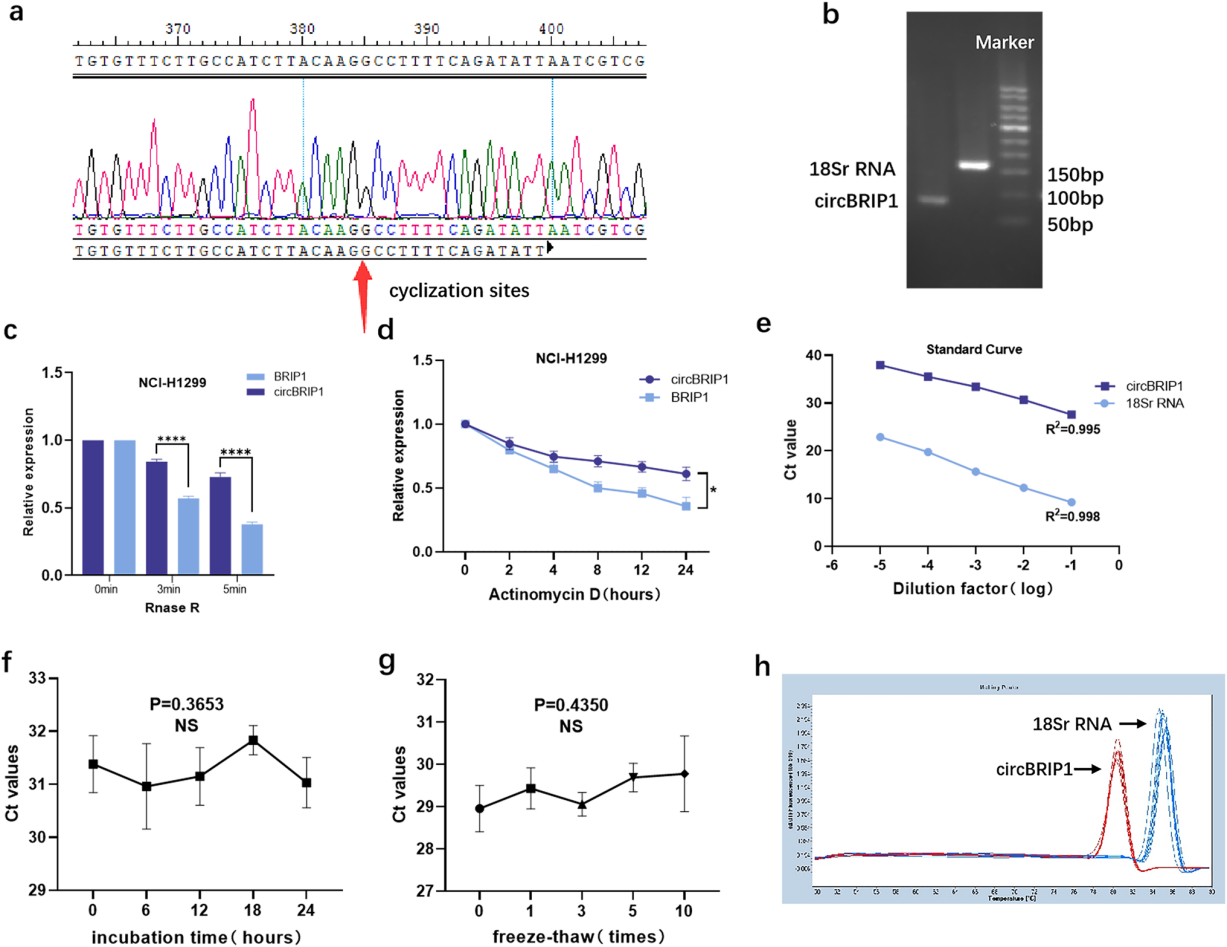
Characteristics of circBRIP1. **a** Sanger sequencing to identify cyclization sites; **b** qRT-PCR product length of circBRIP1 was confirmed by agarose gel electrophoresis (95 bp); **c** stability of circBRIP1 confirmed by RNase R digestion assay; **d** Actinomycin D test demonstrated the half-life of circBRIP1 in NCI-H1299 cell; **e** standard curves of plasma circBRIP1 and 18S in a tenfold serial dilution to demonstrate the linearity; **f, g** stability of circBRIP1 under room temperature incubation time or multiple freeze–thaw cycles; **h** specificity of PCR products by the melting curve

The primers were designed according to the cyclization sites shown in the database, and the products were sequenced by Sanger after qRT-PCR, and the sequencing results proved cyclization sites consistent with those in the database (Fig. 2a). In addition, 2% agarose gel electrophoresis confirmed that the electrophoretic band size of the qRT-PCR product was 95 bp (Fig. 2b), which matched the size of the product boosted by the primer, verifying the accuracy of the product. Then, to verify the stability of circBRIP1, actinomycin D and RNase R assays were used to detect the expression of circBRIP1 under specific treatment times. As shown in the figure, the decrease in the relative expression of BRIP1 is almost twice that of circBRIP1, indicating that its stability met the requirements (Fig. 2c, d).

Next, to investigate whether the method to detect plasma circBRIP1 expression levels (qRT-PCR) could be used for clinical analysis, a comprehensive evaluation was performed. After comparing several internal reference genes (18S, GAPDH, β-actin, β2M, Tub, and RPII), it was found that 18S had the lowest coefficient of variation (CV) (Table 1), so 18S was chosen as the internal reference. First, the assay precision of circBRIP1 was determined using mixed plasma, and the intra- and inter-batch coefficients of variation of both 18 s and circBRIP1 performed well (Table 2). Second, by multiplying the cDNA dilution of NSCLC cells, the regression equation of the standard curve of circBRIP1 expression level was derived as *y* = − 2.558*x* + 25.355, *R*2 = 0.995 (Fig. 2e), which demonstrates good linearity. Then, the mixed plasma of NSCLC patients was left at room temperature for 0, 6, 12, 18 and 24 h, and the other group of mixed plasma was repeatedly freeze–thawed for 0, 1, 3, 5 and 10 times. It can be seen that the CT values of circBRIP1 did not change significantly even if the external conditions were changed (Fig. 2f, g), which demonstrates that circBRIP1 has good stability. In addition, the single peak of qRT-PCR solubility curve was specific (Fig. 2h), indicating the specificity of the method. In conclusion, plasma circBRIP1 is consistent with the characteristics of circRNA, and qRT-PCR is a reliable method to detect its expression level.

**Table 1:** Coefficients of variation of several internal reference genes (*n* = 6)

Reference	Mean ± SD	CV%
18S	12.409 ± 0.74	5.96
GAPDH	27.597 ± 1.96	7.11
β-Actin	23.819 ± 2.45	10.31
β2M	22.931 ± 2.80	12.21
Tub	37.910 ± 3.06	8.06
RPII	32.155 ± 2.33	8.52

**Table 2:** Intra-assay CV and the inter-assay CV of circBRIP1

	circBRIP1	18S rRNA
Intra-assay CV, %	2.92%	2.98%
Inter-assay CV, %	4.46%	4.73%

### Plasma circBRIP1, CEA, SCC and Cyfra21-1 expression

**Fig. 3:**
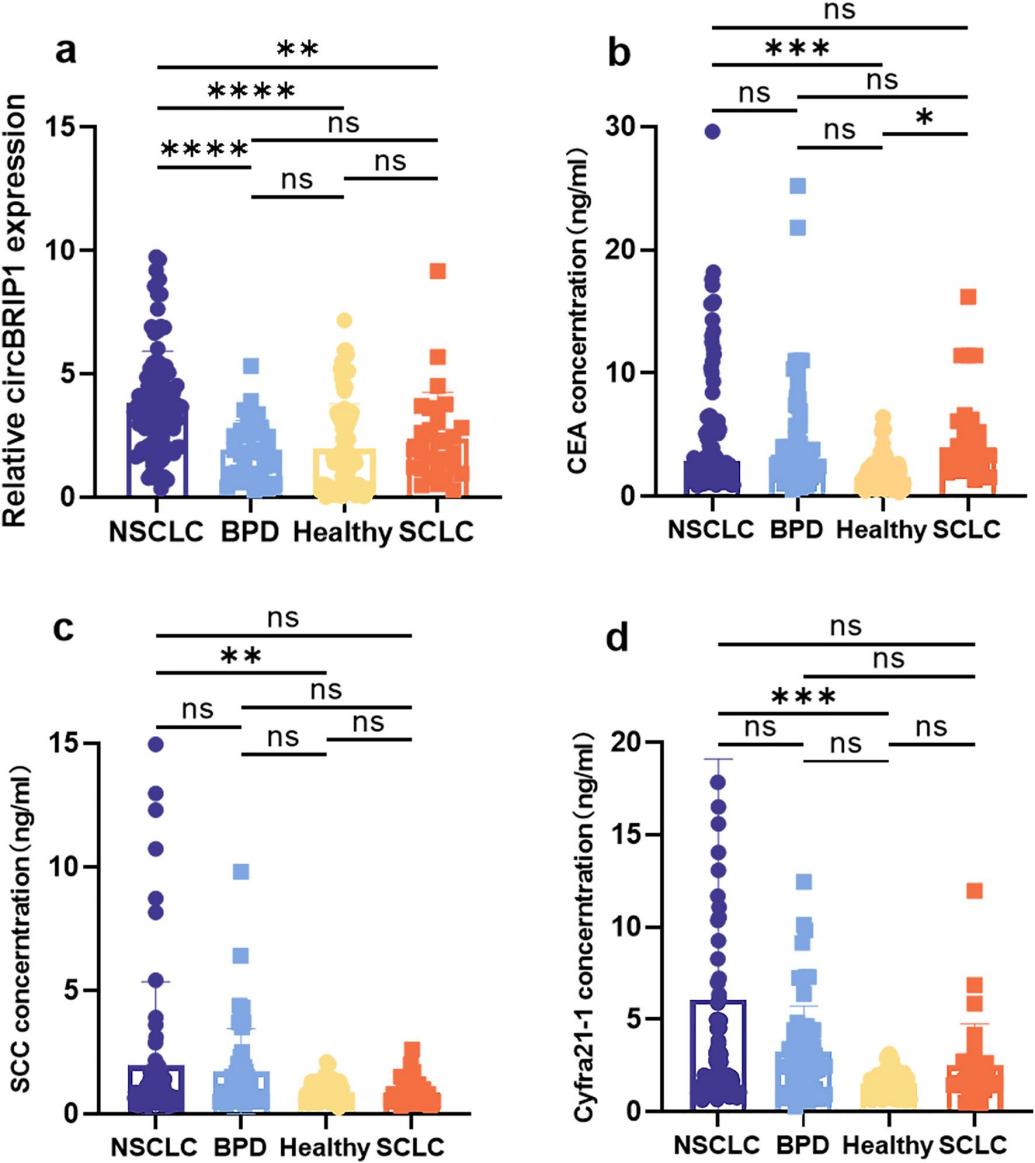
Plasma circBRIP1, CEA, SCC and Cyfra21-1 expressions. **a** Expressions of plasma circBRIP1 levels in NSCLC patients (*n* = 100), BPD patients (*n* = 40) and healthy individuals (*n* = 100), SCLC patients (*n* = 40); **b** expressions of plasma CEA levels; **c** expressions of plasma SCC levels; **d** expressions of plasma Cyfra21-1 levels

Known traditional markers for diagnosing NSCLC can better distinguish NSCLC from healthy individuals. qRT-PCR was performed to verify whether plasma circBRIP1 could be utilized as a diagnostic marker to distinguish patients with more types of lung lesions from healthy individuals. We collected a large number of plasma samples, including 100 patients with NSCLC, 40 patients with BPD, 30 patients with SCLC, and 100 healthy subjects. qRT-PCR results illustrated that compared with traditional markers such as CEA, SCC, and Cyfra21-1 (Fig. 3b–d), circBRIP1 expression levels were not only significantly higher than those of BPD and healthy individuals, but could also significantly distinguish NSCLC patients from BPD (*P* < 0.001) and healthy individuals (*P* < 0.001) (Fig. 3a). Moreover, surprisingly, circBRIP1 was also significant in distinguishing NSCLC from SCLC patients (Fig. 3a, *P* = 0.002).

### Evaluation of single and combined diagnostic models of plasma circBRIP1 on the clinical diagnostic value of NSCLC

**Fig. 4:**
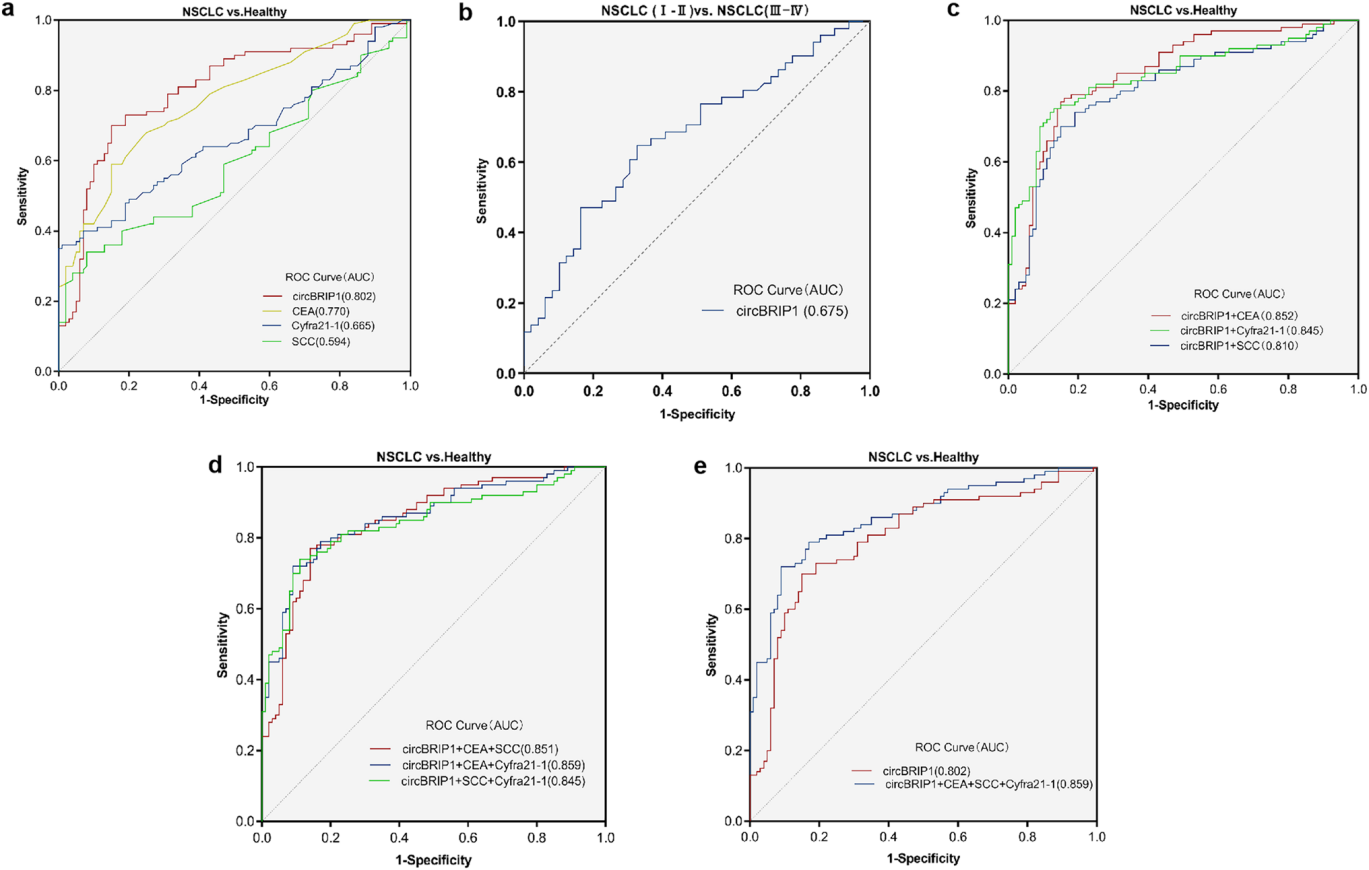
Evaluation of circBRIP1's diagnostic utility in NSCLC. **a** ROC analysis of independent use of plasma circBRIP1, CEA, SCC and Cyfra21-1 in differentiating NSCLC patients (*n* = 100) from healthy donors (*n* = 100); **b** ROC analysis of early and advanced stages of NSCLC; **c–e** ROC analysis of joint diagnostic efficacy of plasma circBRIP1, CEA, SCC, and Cyfra21-1 in distinguishing NSCLC patients with healthy individuals.*P < 0.05, **P < 0.01, ***P < 0.001, ****P < 0.0001 was considered significant, NS (*P* > 0.05) means no statistically difference. ROC, receiver operating characteristic

**Fig. 5:**
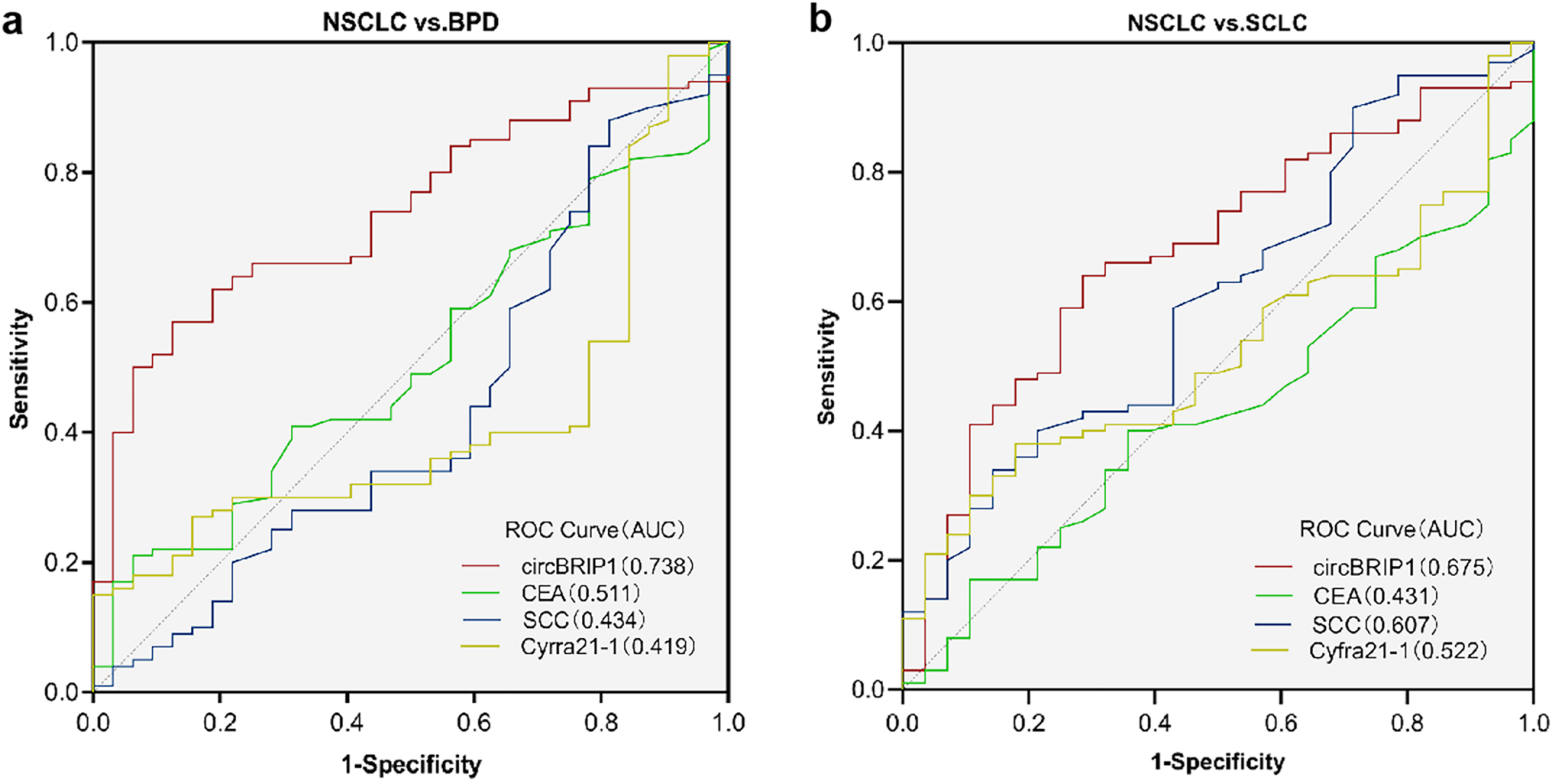
Evaluation of circBRIP1's diagnostic utility in NSCLC. **a** ROC analysis of independent use of plasma circBRIP1 in differentiating NSCLC patients from BPD; **b** ROC analysis of independent use of plasma circBRIP1 in differentiating NSCLC patients from SCLC. *P < 0.05, **P < 0.01, ***P < 0.001, ****P < 0.0001 was considered significant, NS (*P* > 0.05) means no statistically difference. ROC, receiver operating characteristic

CircBRIP1 may be used as a biomarker for NSCLC due to its strong connection with the disease and high expression. However, although plasma circBRIP1 has the molecular properties of a tumor marker, its diagnostic efficiency remains to be investigated. Therefore, we evaluated its clinical diagnostic value. One of the most important criteria for judging circBRIP1 as a unique diagnostic and predictive biomarker is how well it performs diagnostic tests when compared to other well-established lung cancer indicators, including CEA, SCC, and Cyfra21-1. According to the ROC curve, circBRIP1's AUC was 0.802, greater than that of Cyfra21-1 (0.665), SCC (0.594), and CEA (0.770) (Fig. 4a). In addition, we separated NSCLC patients into early (I–II) and late (III–IV) groups using the ROC analysis. AUC of 0.675 in Fig. 4b, which is higher than 0.5, suggests that circcBRIP1 may be able to discriminate between early stage and late-stage lung cancer. When circBRIP1 was combined with other traditional markers, its AUC was higher than when it was used alone. The combination of circBRIP1, CEA, SCC, and Cyfra21-1 had the highest AUC (0.859) (Fig. 4c–e). In addition, the AUC of circBRIP1 was 0.738 in distinguishing NSCLC from BPD and 0.675 in distinguishing NSCLC from SCLC, both of which were higher than the AUC of CEA, SCC, and Cyfra21-1 single diagnosis (Fig. 5a, b). Subsequently, sensitivity (SEN), specificity (SPE), total accuracy (ACCU), positive predictive value (PPV) and negative prediction value (NPV) of single diagnostic models and combined diagnostic models were discussed through logistic regression analysis. CircBRIP1 had a high SEN (0.83) for the diagnosis of NSCLC and healthy subjects, and its indices were improved to different degrees when combined with other markers, among which the sensitivity of circBRIP1 in combination with CEA, SCC and Cyfra21-1 reached the maximum at 0.95 (Table 3). Since Fig. 4e shows that circBRIP1 had a higher AUC (0.738) in differentiating NSCLC and BPD, therefore, the sensitivity of the circBRIP1 diagnosed with NSCLC and BPD was also analyzed, among which the four markers had greater sensitivity in joint diagnosis, 0.83 (Table 4). The above results show that circBRIP1 has a high AUC for a single diagnosis, and a combined diagnosis can improve the diagnostic efficiency. Therefore, circBRIP1 is more accurate in terms of diagnosis than conventional tumor markers, and the combined diagnosis offers better sensitivity for NSCLC.

**Table 3:** Use the expression levels of circBRIP1, CEA, SCC, Cyfra21-1 to distinguish NSCLC patients from healthy donors

	SEN	SPN	ACCU	PPV	NPV	
circBRIP1	0.83 (83/100)	0.58 (58/100)	0.71 (141/200)	0.66 (83/125)	0.77 (58/75)	
CEA	0.39 (39/100)	0.94 (94/100)	0.67 (131/200)	0.87 (39/45)	0.61 (94/155)	
SCC	0.28 (28/100)	0.98 (96/100)	0.62 (124/200)	0.88 (28/32)	0.57 (96/168)	
Cyfra21-1	0.41 (41/100)	0.87 (87/100)	0.64 (128/200)	0.76 (41/54)	0.60 (87/146)	
circBRIP1 + CEA	0.90 (90/100)	0.54 (54/100)	0.72 (144/200)	0.66 (90/136)	0.84 (54/64)	
circBRIP1 + SCC	0.90 (90/100)	0.55 (55/100)	0.73 (145/200)	0.67 (90/135)	0.85 (55/65)	
circBRIP1 + Cyfra21-1	0.92 (92/100)	0.51 (51/100)	0.72 (143/200)	0.65 (92/141)	0.86 (51/59)	
circBRIP1 + CEA + SCC	0.92 (92/100)	0.51 (51/100)	0.72 (143/200)	0.65 (92/141)	0.86 (51/59)	
circBRIP1 + CEA + Cyfra21-1	0.94 (94/100)	0.51 (51/100)	0.73 (146/200)	0.66 (94/143)	0.89 (51/57)	
circBRIP1 + SCC + Cyfra21-1	0.93 (93/100)	0.47 (47/100)	0.70 (140/200)	0.64 (93/146)	0.87 (47/54)	
circBRIP1 + CEA + SCC + Cyfra21-1	0.95 (95/100)	0.44 (44/100)	0.70 (139/200)	0.63 (95/151)	0.90 (44/49)

**Table 4:** Use the expression levels of circBRIP1, CEA, SCC, Cyfra21-1 to distinguish NSCLC patients from BPD

	SEN	SPN	ACCU	PPV	NPV
circBRIP1	0.56 (56/100)	0.88 (34/40)	0.65 (91/140)	0.92 (56/61)	0.44 (35/79)
CEA	0.39 (39/100)	0.70 (28/40)	0.48 (67/140)	0.76 (39/51)	0.31 (28/89)
SCC	0.28 (28/100)	0.55 (22/40)	0.36 (50/140)	0.61 (28/46)	0.23 (22/94)
Cyfra21-1	0.41 (41/100)	0.2 (8/40)	0.35 (49/140)	0.56 (41/73)	0.12 (8/67)
circBRIP1 + CEA	0.76 (76/100)	0.63 (25/40)	0.72 (101/140)	0.72 (76/106)	0.71 (25/35)
circBRIP1 + SCC	0.70 (70/100)	0.45 (18/40)	0.63 (88/140)	0.63 (70/112)	0.64 (18/28)
circBRIP1 + Cyfra21-1	0.73 (73/100)	0.20 (8/40)	0.58 (81/140)	0.59 (73/124)	0.50 (8/16)
circBRIP1 + CEA + SCC	0.80 (80/100)	0.30 (12/40)	0.66 (92/140)	0.67 (80/120)	0.60(12/20)
circBRIP1 + CEA + Cyfra21-1	0.81 (81/100)	0.23 (9/40)	0.64 (90/140)	0.72 (81/112)	0.32(9/28)
circBRIP1 + SCC + Cyfra21-1	0.77 (77/100)	0.15 (6/40)	0.59 (83/140)	0.69 (77/111)	0.21(6/29)
circBRIP1 + CEA + SCC + Cyfra21-1	0.83 (83/100)	0.15 (6/40)	0.64 (89/140)	0.71 (83/117)	0.26(6/23)

### Correlation of plasma circBRIP1 with clinicopathological parameters and dynamic detection

**Fig. 6:**
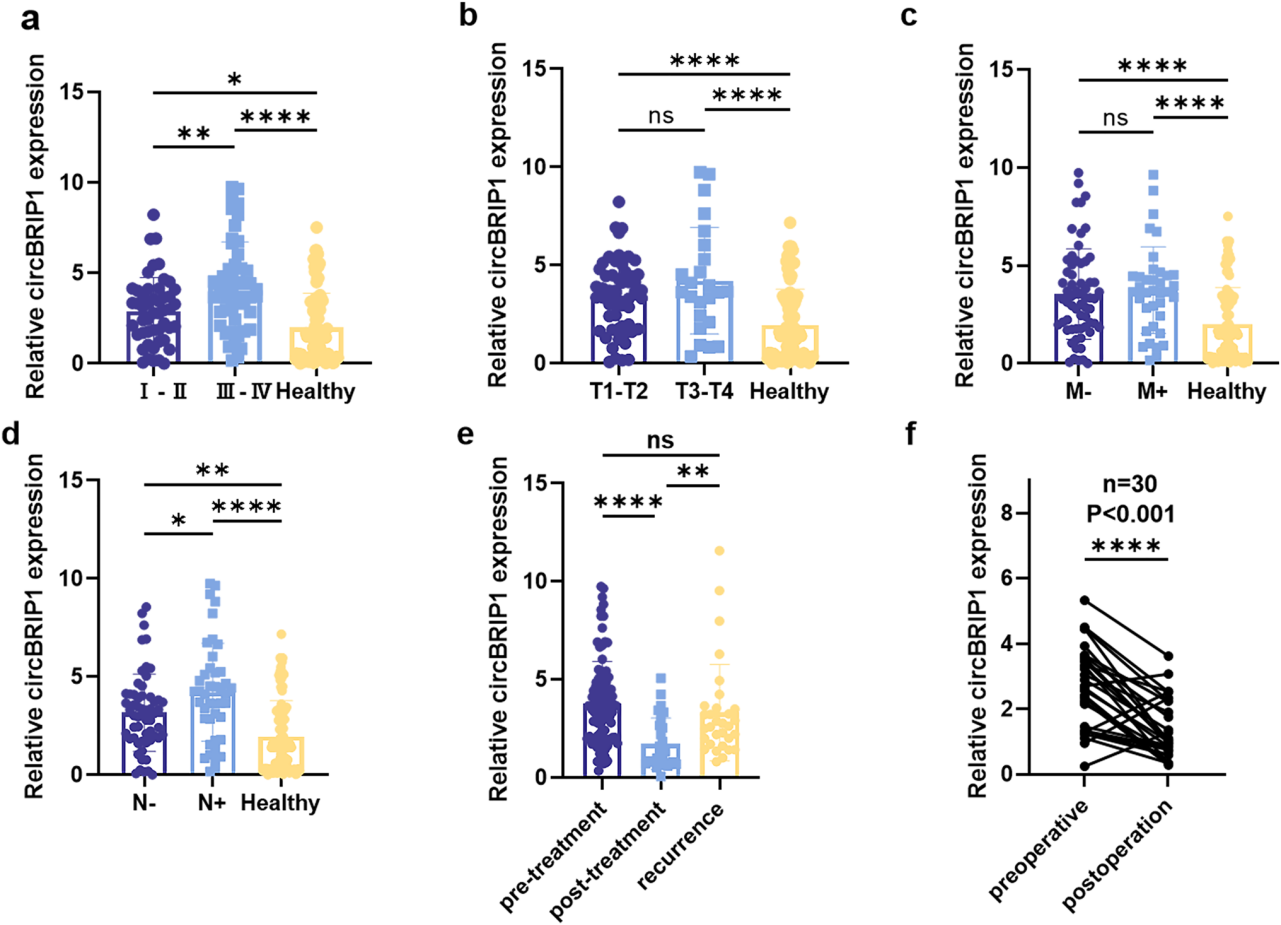
Association between circBRIP1 expression levels and clinicopathologic features of NSCLC patients. **a** circBRIP1 expression in plasma of stage I–II NSCLC patients (*n* = 47), stage III–IV patients (*n* = 53), and healthy individuals (*n* = 100); **b** circBRIP1 expression in plasma in different stages of the depth of tumor invasion and healthy individuals (T1–T2: *n* = 73, T3–T4: *n* = 27, healthy individuals: *n* = 100);** c** circBRIP1 expression in plasma of NSCLC patients with (*n* = 26) or without distant metastasis (*n* = 74); **d** circBRIP1 expression in plasma in NSCLC patients with (*n* = 30) or without lymph node metastasis (*n* = 70); **e** circBRIP1 expression in plasma in 30 pre-treatment patients、30 post-treatment patients and 30 recurrence patients; **f** circBRIP1 expression in plasma in the 30 NSCLC patients before and after the operation

To analyze plasma circBRIP1 expression as well as its clinical value, we examined the relationship between circBRIP1 expression and clinicopathological features. Table 5 summarizes the pathological characteristics of 100 NSCLC patients (Table 5). The 100 NSCLC patients were split into high and low expression groups based on the critical value of plasma circBRIP1. The Chi-square test confirmed that plasma circBRIP1 expression is linked with tumor differentiation, TNM stage, T-stage, lymph node metastasis, and distant metastasis (Table 5). Further examination of clinical features with significant correlation revealed that plasma circBRIP1 expression levels increased significantly with disease progression and were strongly linked with TNM stage and lymph node metastasis (Fig. 6a–d).

**Table 5:** Clinicopathological analysis of circBRIP1 (*n* = 100)

Parameter	circBRIP1		Total	*P* value
	Low expression (*n* = 50)	High expression (*n* = 50)		
*Age(years)*				0.542
≤ 60	22	19	41	
> 60	28	31	59	
*Gender*				0.414
Male	28	32	60	
Female	22	18	40	
*Histological type*				0.790
Adenocarcinoma	42	41	83	
Squamous-cell carcinoma	8	9	17	
*Differentiation*				0.001***
Well or moderate	39	23	62	
Poor or undifferentiattion	11	27	38	
*TNM stage*				0.003**
I + II	31	16	47	
III + IV	19	34	53	
*Tumor size (cm)*				0.338
≤ 5	46	43	89	
> 5	4	7	11	
*T stage*				0.043*
T1–T2	41	32	73	
T3–T4	9	18	27	
*Lymphatic metastasis*				0.001***
Negative	46	24	70	
Positive	4	26	30	
*Distant metastasis*				0.009**
Negative	41	29	74	
Positive	9	21	26	
*Pleural invasion*				0.832
Negative	34	33	67	
Positive	16	17	33	
*Perineural invasion*				0.240
Negative	48	45	93	
Positive	2	5	7	
*Vessel carcinoma embolus*				0.539
Negative	32	29	61	
Positive	18	21	39	
*Airway dispersal*				0.134
Negative	43	37	80	
Positive	7	13	20	
*CEA*				0.041*
Negative	35	25	60	
Positive	15	25	40	
*SCC*				0.171
Negative	40	34	74	
Positive	10	16	26	
*Cyfra21-1*				0.009**
Negative	41	29	60	
Positive	9	21	40	
*CA199*				0.812
Negative	38	39	77	
Positive	12	11	23	
*Serum ferritin*				0.680
Negative	30	32	62	
Positive	20	18	38

To investigate whether the plasma expression levels of circcBRIP1 are associated with tumor dynamics monitoring, by comparing 30 recurrent patients, 30 chemotherapy patients and 30 pairs of pre-pairing post-operative patients, it can be found that the expression level of plasma circBRIP1 decreased by half after chemotherapy and increased relatively after recurrence (Fig. 6e). In addition, the plasma circBRIP1 expression level of postoperative patients was significantly lower compared with that at the initial diagnosis (Fig. 6f). It can be seen that circBRIP1 can be used for dynamic monitoring. In conclusion, we suggest that plasma circBRIP1 can be employed as a novel marker for dynamic monitoring of NSCLC tumors, and its high expression is connected with tumor progression.

### Exploration of the downstream and function of CircBRIP1

**Fig. 7:**
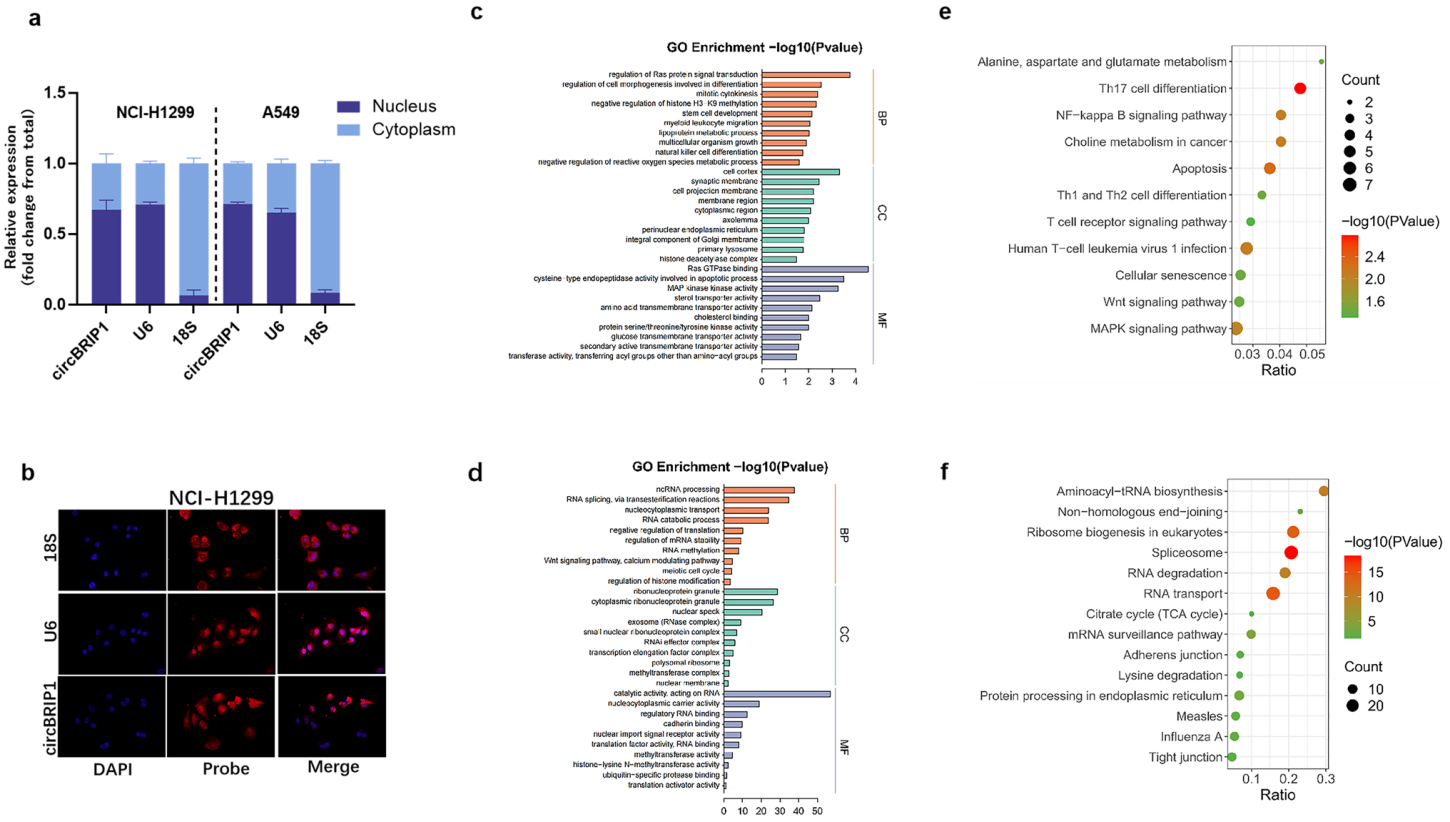
Downstream regulatory network prediction of circBRIP1. **a** Nuclear and cytoplasmic RNA separation text was subjected on NCI-H1299 and SPC-A1 for the identification of circBRIP1; **b** FISH assay of circBRIP1 in SPC-A1 cells; **c, e** GO and KEGG analyzed the CE mechanism of circRNA–miRNA–mRNA regulatory network; **d, f** GO and KEGG analyzed the RBP mechanism of circRNA–RBP regulatory network

The above provides a valid basis for circBRIP1 as a biomarker, and next, we want to look into how circBRIP1 works biologically to advance NSCLC. FISH and nucleoplasm separation experiments showed that circBRIP1 was distributed in both cytoplasm and nucleus, but was more predominant in the nucleus (Fig. 7a, b). CircBRIP1 might contribute to the development of NSCLC mainly through the RNA-binding proteins (RBP) mechanism and may also act as a competing endogenous RNAs (ceRNAs). Therefore, we predicted the circRNA–miRNA–mRNA axis using databases (circAltas, circBank, starBase) and databases (MirPathDB, miRWalk). We next predicted the RBPs of the circBRIP1 connected downstream using the database catRAPID.

The predicted mRNA and RBP were subsequently subjected to bioinformatics analysis. According to a gene ontology study, target genes enriched by the ceRNA mechanism were strongly correlated with Ras GTPase-binding (Fig. 7c), and the target genes predicted by the RBPs mechanism were substantially correlated with catalytic activity (Fig. 7d), both of which were correlated with molecular function. According to KEGG analysis, the ceRNA mechanism predicted target genes were strongly connected with Th17 cell differentiation (Fig. 7e) and the RBPs mechanism proposed target genes were highly associated with the spliceosome (Fig. 7f). The above results suggest circBRIP1's potential involvement in NSCLC progression through its potential targeting of downstream mRNA or RBPs, offering suggestions for additional research into the biological mechanisms of circBRIP1 in the development of NSCLC.

## Discussion

Lung cancer is the primary reason for cancer-related fatalities globally (Cancer of the Lung 2021). Among them, NSCLC, the most prevalent type of lung cancer (Ettinger et al. 2013), has different pathological features and a poor overall prognosis (Verdecchia et al. 2007). One of the main causes of the poor outcomes of NSCLC patients is the lack of effective biomarkers for diagnosis and targeted therapy (Chen et al. 2014a), and early diagnosis of NSCLC can effectively improve the prognosis. CEA has been frequently employed as a tumor marker for the diagnosis of NSCLC, but many tumors can produce CEA, and false positives can be produced in some benign tumors and non-neoplastic diseases (Hao et al. 2019), and smoking can also cause false positives, so its specificity is low (Stockley et al. 1986; Booth et al. 1973). Although the specificity of SCC for the diagnosis of NSCLC is high, its sensitivity is lower than that of CEA (Nikliński et al. 1992; Moro et al. 1995), and Cyfra21-1 lacks organ and site specificity and generally has to be tested in combination with traditional tumor markers to improve the confirmation of NSCLC (Li et al. 2012). For patients with NSCLC, the chances of recurrence and metastasis after early surgery remain high, and sensitivity to radiotherapy is relatively low. While molecularly targeted therapies and immunotherapy have significantly improved the effectiveness of treatment over the past two decades, a large proportion of late-term NSCLCs have grown in tolerance to these therapies and finally advanced (Wang et al. 2021). As a result, it is critically necessary to develop a method that is both efficient and trauma-free for lung cancer early detection and screening. In contrast, serological assays have the advantages of high efficiency, high sensitivity, convenience, easy availability of specimens and low invasiveness (Zhang et al. 2012), so screening for tumor markers has been the focus of early diagnostic studies in NSCLC. This has also prompted us to search for biomarkers with improved sensitivity and specificity. While circRNAs have a series of abundant and stable biological activities and can be widely expressed. It also has biological functions such as participating in the regulation of various physiological and pathological pathways, altering the tumor microenvironment, and regulating tumor cell metabolism (Yu et al. 2019). Therefore, circRNAs have great potential as new biomarkers as well as in promoting the biological progression of tumors.

In this work, we demonstrated for the first time that NSCLC tissues, cells, and plasma had considerably greater levels of circBRIP1 expression than surrounding normal tissues, cells, and normal human plasma. Next, the cyclic properties of circBRIP1 were verified by room temperature placement, repeated freeze–thaw, actinomycin D and RNase R assays. qRT-PCR was utilized as a method to measure its expression level, and we successively verified that its linearity, precision, stability and specificity met the requirements. Then, large samples of plasma were carried out after qRT-PCR and it was found that not only was the level of expression in the plasma of NSCLC patients significantly higher than in BPD and healthy people, but it was also able to significantly differentiate between NSCLC patients and BPD and healthy individuals, as well as to a certain extent distinguish between NSCLC and SCLC. Finally, ROC curve analysis revealed that the diagnostic effectiveness of circBRIP1 individually was significantly higher than that of CEA, SCC and Cyfra21-1. In addition, it is the development trend of tumor diagnosis from single to combined markers, and the analysis revealed that the sensitivity and specificity of circBRIP1 combined with multiple tumor markers were considerably greater than those of single biomarkers, which is expected to further improve its accuracy in the diagnosis, prognosis and prediction of NSCLC. In conclusion, circBRIP1 is a promising biomarker for NSCLC.

Since high expression of circBRIP1 was strongly and positively connected with clinical TNM stage and lymph node metastasis of NSCLC, which corresponded to biological behaviors such as cell proliferation and invasion, we hypothesized that circBRIP1 contributed in the formation of NSCLC. Therefore, we first determined the subcellular localization of circBRIP1 by FISH and nucleoplasm separation assay. CircBRIP1 was more localized in the nucleus, indicating that circBRIP1 mainly interacts with RBP and may also act as a miRNA sponge. In addition, bioinformatic analysis of the circRNA–miRNA–mRNA regulatory axis of circBRIP1 in NSCLC and the downstream RBP showed that among these predictable targets, hsa-miR-526b-3p, a miR that inhibits PD-L1, can inhibit NSCLC development and regulate cell cycle, migration, invasion, apoptosis, tumor chemosensitivity and host antitumor immune response, and its ectopic expression may improve the prognosis of NSCLC patients (Shadbad et al. 2022). Has-miR-302a-3p overexpression reduces the expansion of the stomach cancer cell line SGC-7901 and promotes apoptosis by targeting the negative control of VEGFA (vascular endothelial growth factor A) expression (Yang and Deng 2019). AGO1 may regulate HCC (hepatocellular carcinoma) cell growth and metastasis through the TGF-β pathway (Wang et al. 2018b). By binding to the processor protein DGCR8 and favorably controlling the pri-miR222/6 pathway in a m221A-dependent manner, METTL3 (methyltransferase-like 3) may play an oncogenic function in bladder cancer (Han et al. 2019). Moloney leukemia virus 10 (MOV10)-binding circDICER1 controls the angiogenesis of gliomas via miR-103a-3p/miR-382-5p-mediated ZIC4 (Zinc finger of the cerebellum 4) expression change (He et al. 2019). These findings reveal different regulatory networks in different cancer processes, which may involve circBRIP1.

GO and KEGG analyses revealed that circBRIP1 may be associated with tumor immunity. Although the body has several immunosurveillance systems, it is nevertheless challenging to prevent the emergence and development of malignancies. It is difficult for a few tumor cells to trigger the immune system's response. Until the tumor grows to a certain level and exceeds the body's ability of immune response, the tumor cells are capable of escaping. The mechanism of tumor immune escape involves several immune response links. Tumors can avoid immune detection by overexpressing molecules that keep normal peripheral tissues tolerable, including the interaction of the tumor-associated programmed cell death 1 ligand 1 (PDL1) and the immune receptor programmed cell death 1 (PD1) (Chen et al. 2014b). The PD-1/PD-L1-signaling pathway has been shown to have significant efficacy as an immunotherapeutic target for a variety of tumors, including bladder, lung, and pancreatic cancers. In addition, lncRNA and circRNA are crucial for regulating the PD-1/PD-L1 path and are involved in immunotherapy and immune response (Jiang et al. 2021). Elevation of circRNA FAT1 (circFAT1) in squamous cell carcinoma (SCC) combines and controls the positive association between tumor stemness and immune evasion by stimulating the expression of STAT3 (signal transducer and activator of transcription 3). In contrast, circFAT1 significantly enhanced PD-1 blockade immunotherapy by encouraging CD8+ cell infiltration into the tumor microenvironment. It is evident that circFAT1 is clearly essential for regulating tumor stemness and antitumor immunity (Jia et al. 2021).

In addition, the enrichment analysis revealed that circBRIP1 may be involved in NSCLC progression through methylation. Methylation, a crucial form of epigenetic regulation, is the process of reactive methyl groups being transferred to specific molecules under the catalysis of methyltransferases without altering the DNA sequence (Dai et al. 2021). M6A (N6-methyladenosine)-dependent mRNA regulation, the most typical mRNA modification in eukaryotes, is essential for mammals and has a significant impact on a number of biological processes (Geula et al. 2015). M6A modifications have been reported to be widely present in RNA (He and He 2021). M6A-modified circNSUN2 binds YTHDC1 (YTH domain-containing protein 1) and promotes its exit from the nucleus, further binding IGF2BP2 (lnsulin-like growth factor 2 mRNA-binding protein 2), which stabilizes HMGA2 (high mobility group AT-hook 2) mRNA, thus promoting colorectal cancer liver metastasis (Chen et al. 2019). ALKBH5 (alkylation repair homolog 5) promotes invasive spread of gastric cancer by lowering the methylation of lncRNA NEAT1, and it may be a possible target for therapy (Zhang et al. 2019). In addition, whether the mechanism of circBRIP1 involvement in NSCLC progression is related to tumor immunity or methylation needs to be further investigated and validated.

## Supplementary Information

Below is the link to the electronic supplementary material.

Supplementary file1 (DOCX 3280 KB)

## Data Availability

Upon reasonable request, the relevant author will supply the datasets used and/or analyzed for the current work.
