# Supplementary material for: CircBRIP1: a plasma diagnostic marker for non-small-cell lung cancer
Source: J Cancer Res Clin Oncol. 2024 Feb 8;150(2):83. doi: 10.1007/s00432-023-05558-5 (PMC10853360; doi:10.1007/s00432-023-05558-5)
Supplement: Supplementary file 1 — Supplementary file1 (DOCX 3280 KB) [file 432_2023_5558_MOESM1_ESM.docx]

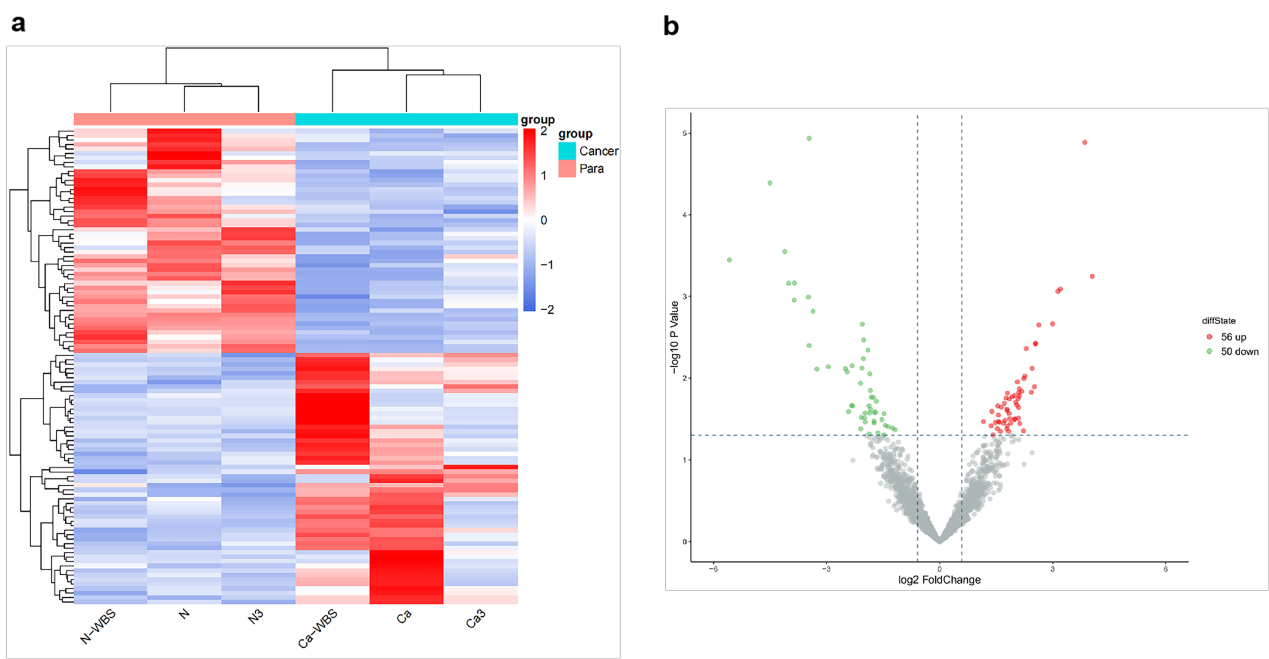


Supplementary **Fig.a** The clustered heatmap showed the differentially expressed circRNAs in 3 pairs of human NSCLC tissues and adjacent normal tissues; **b** The volcano plots of circRNAs expressions. The red and green strips indicate up-regulated and down-regulated circRNAs, respectively.
